# A Prognostic Model of Seven Immune Genes to Predict Overall Survival in Childhood Acute Myeloid Leukemia

**DOI:** 10.1155/2022/7724220

**Published:** 2022-12-05

**Authors:** Yan Luo, Yanpeng Xu, Xue Li, Xiaoqi Shi, Pei Huang, Yan Chen, Zhixu He

**Affiliations:** ^1^Suzhou Medical College of Soochow University, Suzhou 215325, China; ^2^Department of Pediatrics, Affiliated Hospital of Zunyi Medical University, Zunyi 563000, China; ^3^Department of Pediatrics, Guizhou Children's Hospital, Zunyi 563000, China; ^4^Collaborative Innovation Center of Tissue Damage Repair and Regeneration Medicine of Zunyi Medical University, Zunyi, 563003 Guizhou, China

## Abstract

**Background:**

Acute myeloid leukemia (AML) is one of the most common hematological malignancies and accounts for about 20% of childhood leukemias. Currently, immunotherapy is one of the recommended treatment schemes for recurrent AML patients to improve their survival rates. Nonetheless, low remission and high mortality rates are observed in recurrent AML and challenge the prognosis of AML patients. To address this problem, we aimed to establish and verify a reliable prognostic risk model using immune-related genes to improve the prognostic evaluation and recommendation for personalized treatment of AML.

**Methods:**

Transcriptome data and clinical data were acquired from the TARGET database while immune genes were sourced from InnateDB and ImmPort Shared databases. The mRNA expression profile matrix of immune genes from 62 normal samples and 1408 AML cases was extracted from the transcriptome data and subjected to differential expression (DE) analysis. The entire cohort of DE immune genes was randomly divided into the test group and training group. The prognostic model associated with immune genes was constructed using the training group. The test group and entire cohort were employed for model validation. Lastly, we analyzed the potential clinical application of the model and its association with immune cell infiltration.

**Results:**

In total, 751 DE immune genes were differentially regulated, including 552 upregulated and 199 downregulated. Based on these DE genes, we developed and validated a prognostic risk model composed of seven immune genes, *GDF1*, *TPM2*, *IL1R1*, *PSMD4*, *IL5RA*, *DHCR24*, and *IL12RB2*. This model is able to predict the 5-year survival rate more accurately compared with age, gender, and risk stratification. Further analysis showed that CD8^+^ T-cell contents and neutrophil infiltration decreased but macrophage infiltration increased as the risk score increased.

**Conclusions:**

A seven-immune gene model of AML was developed and validated. We propose this model as an independent prognostic variable able to estimate the 5-year survival rate. In addition, the model can also reflect the immune microenvironment of AML patients.

## 1. Introduction

Acute myeloid leukemia (AML) is a malignant clonal disease that arises from abnormal immature myeloid cell proliferation which blocks differentiation. The main clinical features of AML include anemia, bone pain, coagulation dysfunction, and enlargement of liver, spleen, and lymph nodes [1]. Previous reports have noted that about 15%-20% of children with acute leukemia (AL) are diagnosed as AML, and the majority of them are boys [2, 3]. Continued improvement in terms of supportive treatment, chemotherapy regimen, and stem cell transplantation technology have resulted in long-term survival rates of AML patients at about 70% [4–6]. However, about 30% of patients in remission will go on to relapse and the five-year survival rate after recurrence of AML is only 36.1% [7].

Immunotherapy may be one of the important treatment schemes to delay disease recurrence and to improve patients' survival rates. In 2016, Zahler et al. [8] used the anti-CD33 antibody coupled to the drug gemtuzumab ozogamicin (Go) on 14 children who had been treated by bone marrow transplantation. Following treatment, their overall survival rates at 1-year and 5-years were 78% and 61%, respectively [8]. An adult phase 3 trial indicated that the chemotherapy drugs combined with Go significantly improved event-free survival (EFS) of AML patients while the adverse side effects were not aggravated [9]. However, a different study reported that 21 children with recurrent AML, who were treated with allogeneic therapy combined with NK cells, did not significantly improve EFS and overall survival (OS) compared with those without NK cells [10]. The inconsistent response to immunotherapy as reported in various studies may be associated with the heterogeneous nature of AML. Therefore, we believe that there is an urgent need to develop and evaluate a molecular model that can reflect the prognostic outcomes of patients to immunotherapy to justify personalized treatment for AML children.

In the present study, transcriptome data and clinical data of AML patients were first obtained from the TARGET database and applied to establish and verify a reliable prognostic risk model by using DE immune genes. This risk model also clarifies the theoretical foundation for the prognostic evaluation and provides a basis for the development of personalized treatment for AML children.

## 2. Materials and Methods

### 2.1. Material Sources

A total of 1470 transcriptome data sets (62 for the normal group and 1408 for acute myeloid leukemia patients) as of August 21, 2021 were retrieved from the Target database (https://ocg.cancer.gov/programs/target). It is well known that the Target database is a children's tumor database. In addition, the associated clinical parameters were also obtained from the same source. The clinical data excluded any unclear clinical stage and unknown prognostic information but included the following parameters: survival time, survival status, age (range 0-30 years, and median age was 9.9 years old, however, the proportion of AML children whose age ranged from 0 to 18 years old was 95.5%), gender, white blood cell counts (WBC), bone marrow leukemic blast, French-American-British (FAB) category, chromosome karyotype, risk stratification, remission after the first course of treatment (CR1), and remission after the second course of treatment following a recurrence (CR2). The AML subtypes included M0-M7 based on the FAB classification system. This research did not require any approval from an ethics committee, as all data used were publicly available.

### 2.2. Extraction of the Gene mRNA Expression Matrix

The Perl programming language (https://www.perl.org/) was applied to extract the original number of transcripts data for acute myeloid leukemia and normal samples. The expression profile data were then annotated according to the Homo sapiens GRCH38. 95.chr. Gtf. GZ file retrieved from the Ensembl database (https://asia.ensembl.org/index.html). A total of 6194 immune genes were downloaded from the Immport Shared Database (https://www.immort.org/) and InnateDB database (InnateDB) (https://www.innatedb.ca/). Duplicate genes were removed and the mRNA expression profile matrix of 2660 immune genes was extracted from the transcriptome data using Perl script.

### 2.3. Identification of Differentially Expressed Immune Genes

The “edgeR” package of R 4.1.2 software (https://www.r-project.org/) was employed to screen for differentially expressed (DE) immune genes from the expression profile matrix. Statistical significance was set with a threshold of false discovery rate (FDR) <0.5 and log_2_|fold change|(FC) >1. The heatmap and volcano plot were generated with the pheatmap and gplot package of the R 4.1.2 software, respectively.

### 2.4. Functional and Pathway Enrichment Analysis

To explore the biological functions and pathways of the DE immune genes, the “org Hs.Eg.db” and “clusterProfiler” packages were employed for gene ontology (GO) analysis and Kyoto Encyclopedia of Genes and Genomes (KEGG) analysis. The top ten GO biological processes including biological process (BP), cellular component (CC), and molecular function (MF) with marked enrichment were visualized, while the KEGG pathways were visualized based on the top thirty terms. Enrichment of the GO terms and KEGG terms were deemed statistically significant at *P* < 0.05.

### 2.5. Establishment of an Immune Gene Prognostic Risk Model

The survival period and survival status obtained from the patients' clinical records were combined with the mRNA data of DE immune genes from the entire data set and then randomly assigned to 2 cohorts to form the training group and test group by using Rv. Uniform function in the SPSS software (IBM spss 23.0, Armork, New York, USA). Using the DE immune genes within the training set, univariate Cox analysis, and Kaplan Meier analysis were conducted to identify significant DE immune genes that were relevant to establish a reliable prognosis using the “survival” package of the R 4.1.2 software. Further to this, multivariate Cox regression (MCR) analysis was carried out to select the immune genes with more promising prognostic values to establish the model. Lastly, the risk score of samples was measured using the Cox coefficient and gene expression values with the following formula.

Risk score = Coei1^∗^Expi1 + Coei2^∗^Expi2 + ⋯+Coeix^∗^Expix (Coei: the coefficient value, and Expi: the gene expression level (FPKM)) [11, 12].

### 2.6. Determining the Reliability and Independent Prognostic Value for the Risk Model

The median risk score values of the samples were used to delineate the training set into high-risk (HR) and low-risk (LR) groups. The survival curves for the respective groups were drawn with “survminer” within the R package (version 4.1.2); then, time-dependent receiver operating characteristics (ROC) analysis for the overall survival (OS) and the area under the ROC (AUC) were performed using the R package “survivalRoc” to examine the specificity and sensitivity of the prognostic model. AUC prediction values >0.60 were considered acceptable while an AUC prediction value of over 0.75 was considered excellent [13, 14]. To assess the prognostic value of the model, univariate and multivariate Cox analyses were carried out on the prognostic factors selected from the patients' clinical features. Patients were assigned to 2 groups based on age (<10 and ≥10 years old). Patients were assigned to three groups according to WBC ( = <50, 50 − 100 and > = 100). Patients were assigned to three groups according to bone marrow leukemic blast ( = <20, 20 − 80 and > = 80). Heatmaps, survival status scatter plots, and risk score distribution plots between the HR and LR groups were also produced and visualized to assess the model.

Meanwhile, we built a nomograph based on the 7 immune genes in our model using the R packages “rms”, “regplot”, and “survival”, and the constructed nomograph was used to predict the OS of the individual AML patients. If the calibration line was very close to the 45-degree line, which represented the ideal prediction, this indicated that the actual survival was very close to the predicted survival. This analysis validated the good prediction power of the constructed nomogram.

### 2.7. Validating the Prognostic Value of the Risk Model Using the Test and Entire Cohorts

The specificity and sensitivity of the prognostic risk model was validated using the test cohort and the entire cohort. As noted above, survival curves, ROC for OS, AUC, heatmaps, survival status scatter plots, and risk score distribution plots were also employed to assess the model. Further validation was also based on the nomogram and its calibration curve as described above. Meanwhile, univariate and multivariate Cox analyses were carried out to analyze the prognostic factors based on the clinical features.

### 2.8. Clinical Utility of the Model

To verify that the selected immune genes involved in the development of AML were the best indicators for our model's predictive ability for prognostication, we analyzed the prognosis of the entire cohort of patients and the relationship between the model (risk gene level and risk score) and the clinical characteristics of the entire cohort (age, gender, risk stratification, CR2).

### 2.9. Association between the Model and Immune Cell Infiltration

To determine if the model reflected the status of the immune microenvironment in AML, we extracted infiltrated immune cells such as CD4 + T cells, B cells, macrophages, dendritic cells, CD8 + T cells, and neutrophils from the transcriptome data of AML patients using the analysis tool (CIBERPORT) firstly, and then we evaluated the association between the risk score and immune cell infiltration in the entire cohort. The Pearson's correlation coefficient test was employed to assess the correlation between risk score of the model and level of different immune cell types. In addition, we analyzed the difference between the low- and high-risk scores of AML-related immune cells in the entire cohort using “limma”,"survival”, and “survminer R” packages.

## 3. Results

### 3.1. Identification of the DE Immune Genes and Analysis of their Functional and Pathway Enrichment

**Figure 1:**
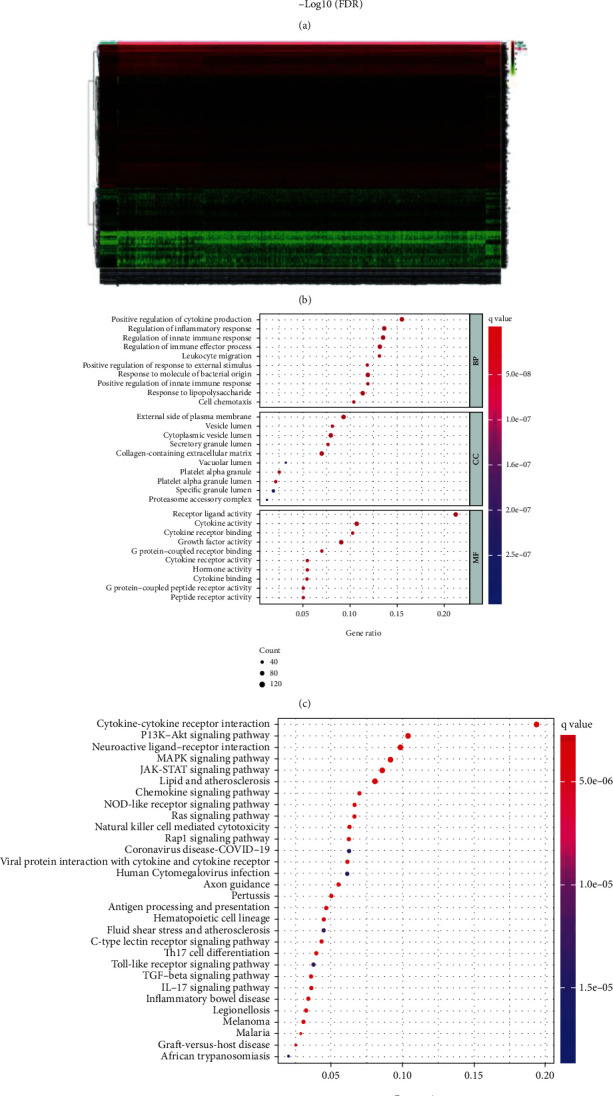
DE immune genes. (a) Volcano plot of the DE genes (the blue line at the top of the figure represented the control group and the pink line represented AML). (b) Heatmap of the DE genes (red and green dots indicated upregulated and downregulated genes, respectively). (c) GO analysis of the DE immune genes for biological process (BP), cellular component (cc), as well as molecular function (MF) terms. (d) KEGG analysis of the DE immune genes.

The transcriptome data sets sourced from the Target database were submitted to Perl and after eliminating duplicates, 2660 immune genes were obtained. Using the “edgeR” software package of R version 4.1.2, a total of 751 immune genes differentially expressed in AML versus normal samples were obtained, including 552 and 199 upregulated and downregulated, respectively. The DE immune genes were evaluated and visualized as a volcano plot and heatmap (Figures 1(a) and 1(b)). To determine the biological function of the DE immune genes, we conducted enrichment analysis on the top 10 GO biological processes and top 30 KEGG pathways. The results demonstrated that the DE immune genes associated with GO were mainly enriched in receptor ligand activity, leukocyte migration, external phase of the plasma membrane, receptor ligand activity, and G protein-coupled receptor binding (Figure 1(c)). The enriched GO functions included several terms which were closely related to the processes involved in AML development but the terms “leukocyte proliferation” and “regulation of hemopoiesis” were not visualizable in Figure 1(c) although also involved in AML.  Figure 1(d) showed the top 30 KEGG pathways including cytokine-cytokine receptor related effects, JAK−STAT signaling pathway, MAPK signaling pathway, PI3K − Akt signaling pathway, and neuroactive ligand receptor related effects while the top 10 KEGG pathways and additional gene information were presented in Table 1.

### 3.2. Establishment of a Prognostic Risk Model from the DE Immune Genes

**Figure 2:**
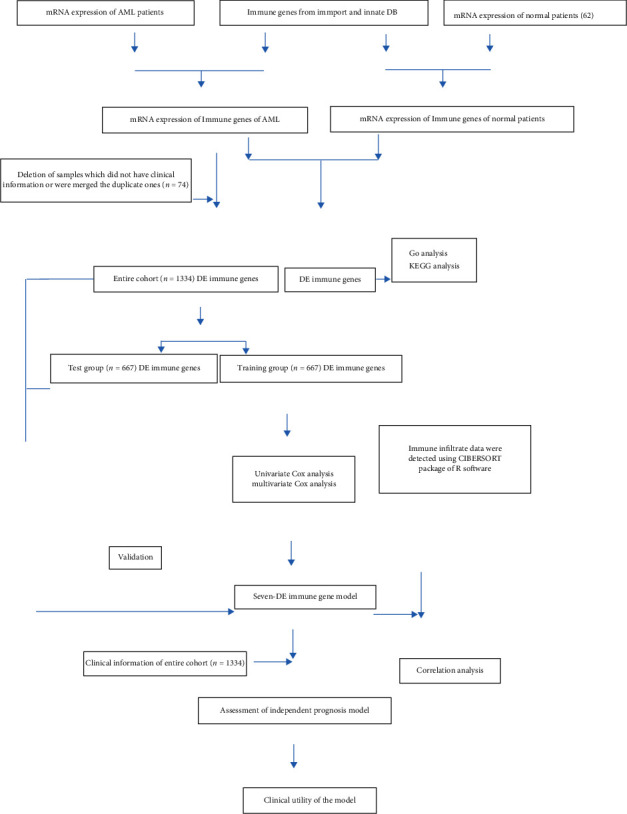
The diagram of experimental analysis process.

To assess the DE immune genes related to AML prognosis in children, we first analyzed 1408 AML samples for the availability of survival status and survival time. A total of 74 samples were removed from the collection as they did not have clinical information or were duplicates of other samples. Then, the remainder 1334 cases were randomly assigned to the training (667 cases) and test (667 cases) groups. The training group was applied to establish the prognostic risk model, while the test group and the entire cohort were employed to verify the model. The workflow for the analysis was shown in Figure 2. In the training group, we screened 114 DE immune genes using univariate Cox regression and Kaplan-Meier analyses for those which markedly correlated with OS in AML patients (Supplementary Table 1). Moreover, MCR analysis was conducted and we identified seven genes that had a greater impact on the OS of patients, which were then applied to establish the prognostic risk model as shown in Table 2.

### 3.3. Verification of the Reliability of the Prognostic Model and Independent Prognostic Value in the Training Group

The seven genes, growth differentiation factor 1 (*GDF1*), tropomyosin 2 (*TPM2*), interleukin 1 receptor 1 (*IL1R1*), proteasome 26S subunit non-ATPase 4 (*PSMD4*), IL-5 receptor alpha (*IL5RA*), Dehydrocholesterol reductase 24 (*DHCR24*), and interleukin-12 receptor beta-2 (*IL12RB2*), were applied to establish the prognostic model. After that, we calculated the risk score of each as follows: Risk score = [expression value of GDF1^∗^(−0.07435)] + (expression value of TPM2^∗^0.279473) + [expression value of IL1R1^∗^(−0.10441)] + (expression value of PSMD4^∗^0.250769) + [expression value of IL5RA^∗^(−0.06511)] + (expression value of DHCR24^∗^0.129822) + (expression value of IL12RB2^∗^0.068248).

**Figure 3:**
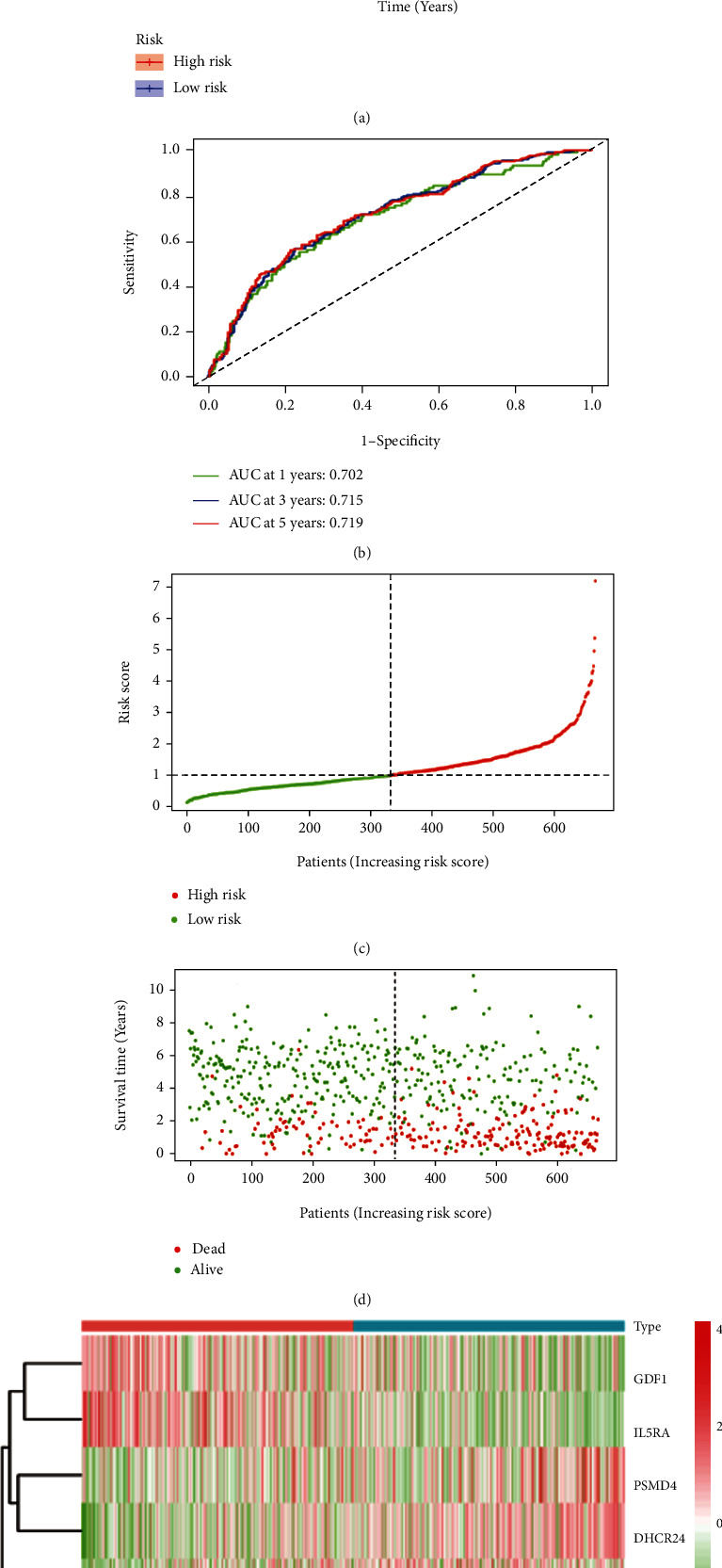
Establishment of the prognostic risk model according to the training group. (a) Overall survival. (b) Time-dependent receiver operating characteristic (ROC) curve analysis. (c) Risk score distribution. (d) Survival status scatter plot. (e) Heatmap of risk genes.

Taking the median risk score as the boundary, the patients were assigned to two groups: HR group and LR group. The survival curve indicated that the OS of patients in the HR group was significantly shortened (*P* < 0.001) (Figure 3(a)). In addition, we used time-dependent ROC to analyze the specificity and sensitivity of our model in predicting the OS and found that the AUC at one, three and five years was 0.702, 0.715, and 0.719, respectively (Figure 3(b)). Moreover, we ranked the risk scores of patients and analyzed their distributions and the dots represented the survival status of each patient and the Heatmap showed the expression of these seven immune genes in the HR and LR groups (Figures 3(c)–3(e)).

**Figure 4:**
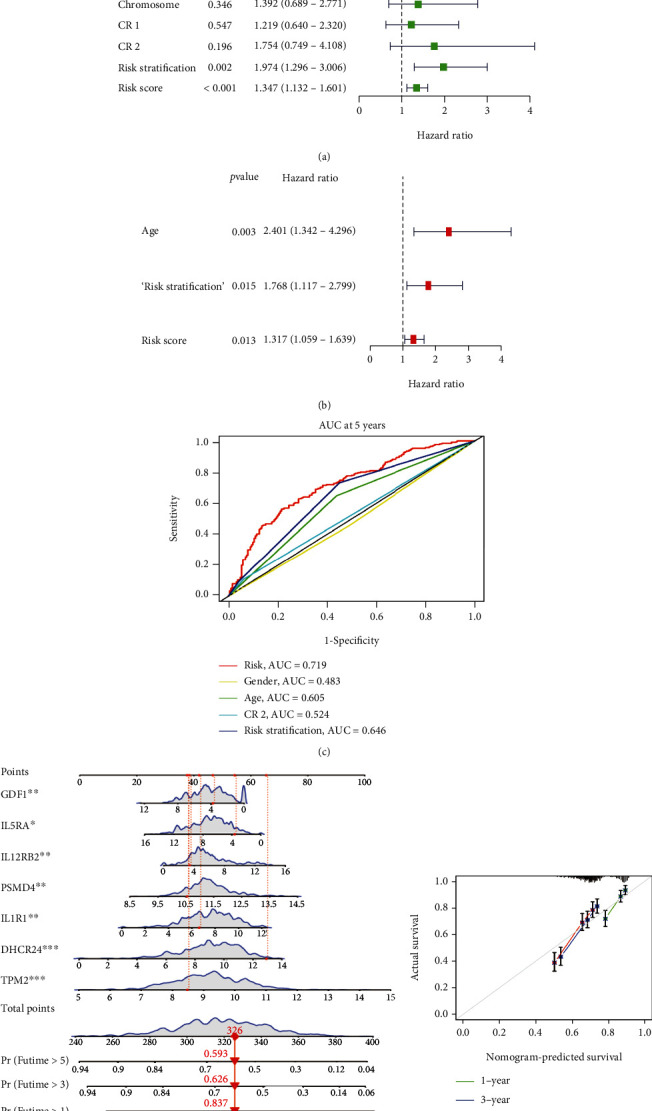
(a) Univariate Cox analysis. (b) Multivariate Cox analysis. (c) ROC cure analysis of prognostic variables in the training group at five years. (d) A nomogram to predict the 1-, 3-, and 5- year OS in AML patients in the training group. (e) Calibration plot of nomogram in the training group.

In terms of the independent prognostic value of the model, both univariate and multivariate Cox analyses showed that age, risk stratification, and the risk score were related to the OS of AML in the training group and proposed that these might be independent prognostic factors (*P* < 0.05) (Figures 4(a) and 4(b)). Then, we further compared these variables and observed that risk score had higher specificity and sensitivity compered to age, gender, risk stratification, and CR2 in predicting the 5-year OS. At 5 years, the AUCs of risk score, age, gender, risk stratification, and CR2 were 0.719, 0.483, 0.605, 0.524, and 0.646, respectively, which demonstrated that the prediction power of risk score was better than the other factors within the training group (Figure 4(c)). In addition, it can be seen from the calibration curve that the constructed nomograph had good predictive performance in terms of predicting the survival rate of the individual patient with AML (Figures 4(d)–4(e)).

### 3.4. Validation and Independent Prognostic Value of the Risk Model in the Test Group and in the Entire Cohort

**Figure 5:**
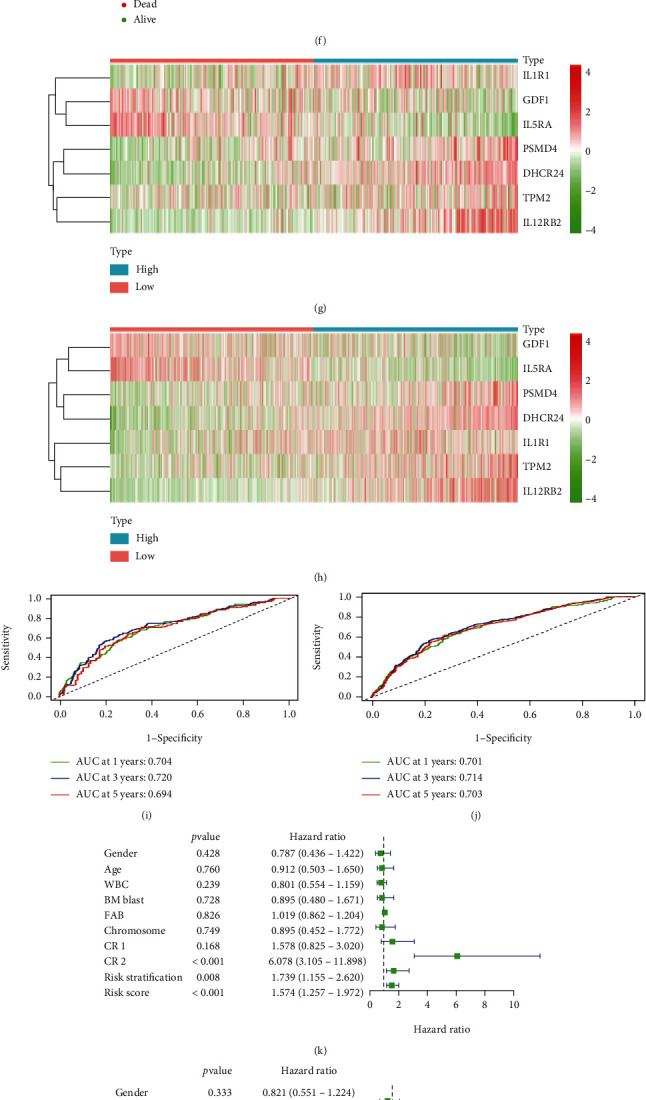
Validation of the prognostic risk model in the test group and entire cohort. (a) OS in the test group. (b) OS in the entire cohort. (c) Risk score distribution in the test group. (d) Risk score distribution in the entire cohort. (e) Survival status scatter plot in the test group. (f) Survival status scatter plot for the entire cohort. (g) Heatmap of risk genes in the test group. (h) Heatmap of risk genes in the entire cohort. (i) ROC curve analysis in the test group. (j) ROC curve analysis in the entire cohort. (k) Univariate Cox analysis in the test group. (l) Univariate Cox analysis in the entire cohort. (m) Multivariate Cox analysis in the test group. (n) Multivariate Cox analysis in the entire cohort. (o) A nomogram to predict 1-, 3-, and 5- year OS in AML patients in the test group. (p) A nomogram to predict 1-, 3-, and 5- year OS in AML patients in the entire cohort. (q) Calibration plot of nomogram in the test group. (r) Calibration plot of nomogram in the entire cohort.

To confirm the accuracy of the risk model, we analyzed the independent prognostic value of the model in the test group and the entire cohort. First, the risk scores of each patient were calculated and then assigned to HR and LR groups according to the median risk score. In the test group, the survival probability was significantly lower in the HR group and the same result was observed for the entire group (*P* < 0.001) (Figures 5(a) and 5(b)). Figures 5(c)–5(h) showed the risk score distribution, survival status, and risk gene expression heatmap in the two cohorts. In addition, the AUCs in the test group at 1, 3, and 5 years were 0.704, 0.720, and 0.694, respectively, and were 0.701, 0.714, and 0.703, respectively, for the entire cohort (Figures 5(i) and 5(j)). Moreover, the univariate and multivariate analyses revealed that CR2 and risk score were related to the prognosis of patients in the test group (*P* < 0.05) (Figures 5(k) and 5(m)). Meanwhile, we found that CR2, risk stratification, and risk score were independent prognostic factors for the entire cohort (*P* < 0.05) (Figures 5(l) and 5(n)). Moreover, when either the test group or the whole cohort data was used, the predictive ability of the constructed nomograph as based on the calibration curve was good in terms of predicting the survival rate of the individual patient (Figures 5(o)–5(r)).

### 3.5. Correlation between the Risk Model and AML within the Entire Cohort

**Figure 6:**
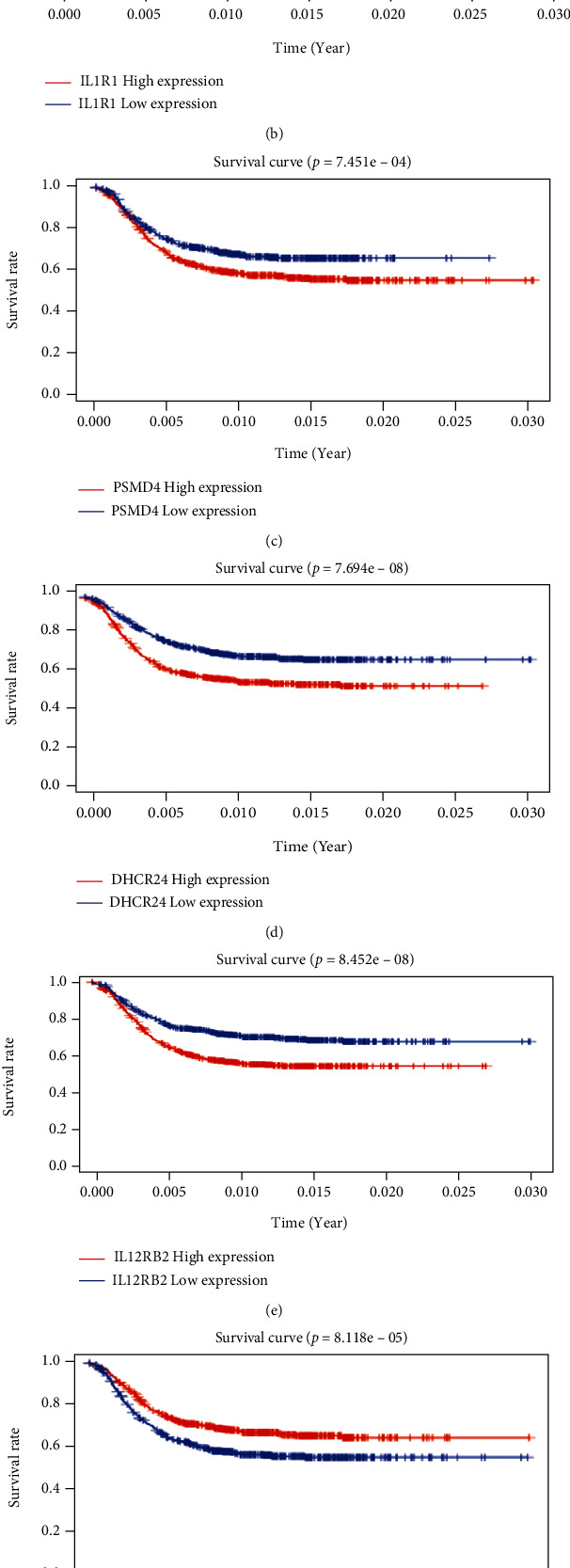
Prognostic value and mRNA expression of the model within the entire cohort. (a)–(g) Relationship between *TPM2*, *IL1R1*, *PSMD4*, *DHCR24*, *IL12RB2*, *IL5RA*, *GDF1*, and OS in AML.

**Figure 7:**
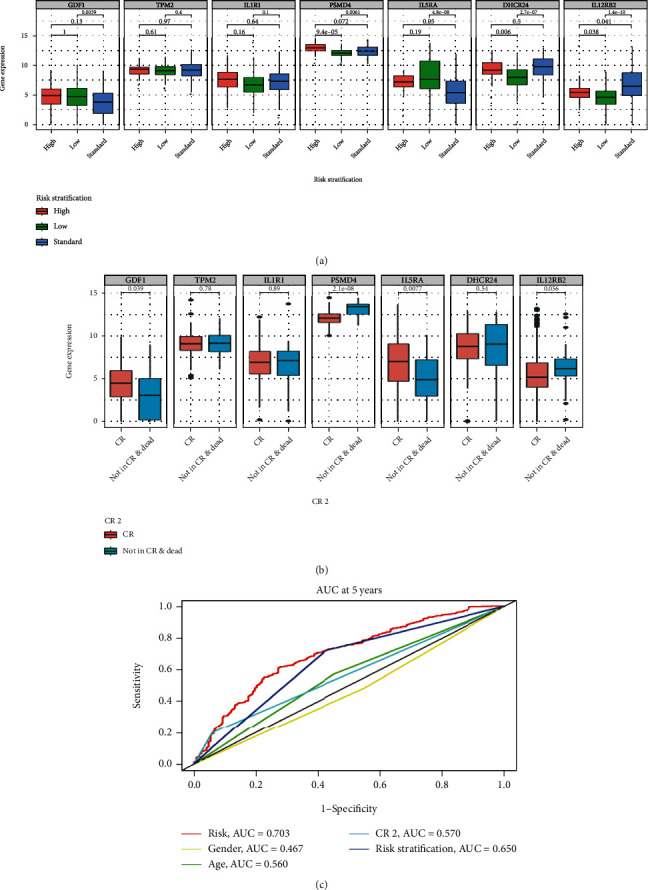
Independent prognostic value of the model for the entire cohort. (a) Relationship between genes in the model and risk stratification. (b) Relationship between genes in the model and CR2. (c) ROC analyses of the prognostic factors.

To assess whether the genes in our model were indeed involved in the development of AML, we analyzed the relationship between their expression and OS in the entire cohort. The findings indicated that all genes were related to OS and that the lower the expression of *IL5RA* and *GDF1*, the worse the prognosis of AML in children while the higher the expression of *TPM2, IL1R1*, *PSMD4*, *DHCR24*, and *IL12RB2*, the worse the prognosis for AML children (Figures 6(a)–6(g)). Then, we further analyzed the relationship between risk genes, risk score, and clinical features. In terms of the association between risk genes and risk stratification, we discovered that the expression levels of *GDF1* and *IL5RA* genes in low-risk AML patients were increased compared to those in HR and standard risk groups, while the expression levels of *PSMD4*, *DHCR24*, and *IL12RB2* genes in low-risk AML patients were lower (Figure 7(a)). As for the relationship between risk genes and CR2, it was observed that expression levels of *IL5RA* and *GDF1* genes were higher in CR2; while the expression level of *PSMD4* was lower (Figure 7(b)). Lastly, we compared the different variables and observed that the specificity and sensitivity of the risk score was higher in predicting 5-year OS when compared to age, gender, CR2, and risk stratification (Figure 7(c)_). These results also suggested that these immune genes be associated with the development of AML in the entire cohort.

### 3.6. Correlation between the Model and Immune Cell Infiltration

**Figure 8:**
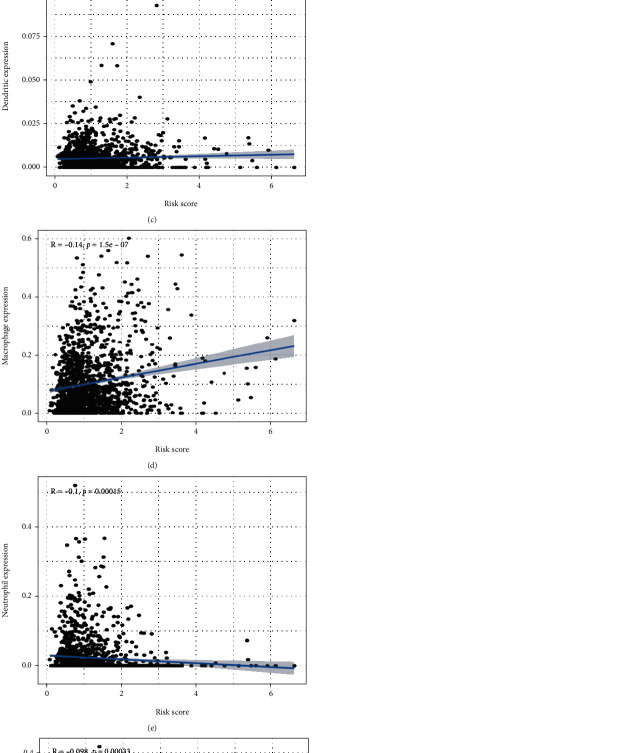
Association analysis between the risk score and immune cell infiltration. (a) B cells. (b) CD4^+^ T cells. (c) Dendritic cells. (d) Macrophages. (e) Neutrophils. (f) CD8^+^ T cells.

**Figure 9:**
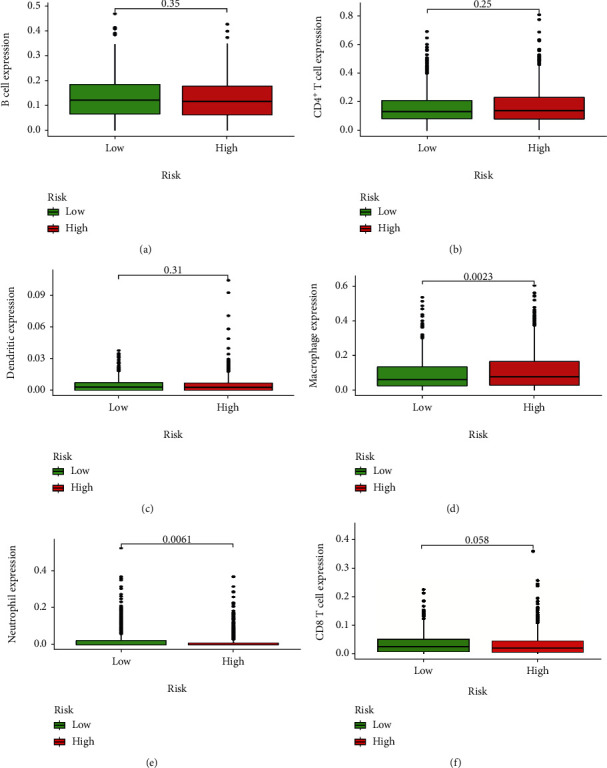
The different expression analysis between the high and low risk score of immune cell infiltration. (a) B cells. (b) CD4^+^ T cells. (c) Dendritic cells. (d) Macrophages. (e) Neutrophils. (f) CD8^+^ T cells.

Within the entire cohort, the risk score was positively correlated with macrophage content and negatively correlated with CD8^+^ T cells and neutrophils (Figures 8(a)–8(f)). In addition, macrophage content was significant higher in the HR group, while the neutrophils were lower. For CD8^+^ T cells, no differences were noted between the HR and LR group (Figures 9(a)–9(f)). These findings demonstrated that the selected immune genes in the model might reflect the immune microenvironment status of AML patients.

## 4. Discussion

AML is a common hematological malignancy in children with chemotherapy and allogeneic stem cell transplantation provided as the standard treatment. Although these treatments continue to be optimized and the 5-year survival rate of children is remarkably increased, the outcome is still not ideal because of the low remission rates and high mortality in patients with recurrent AML [15, 16]. Growing evidence suggests that cells of the immune system could regulate immunoprotein-encoding genes and immunotherapy may be an alternative approach to improve the survival rate of children with recurrent AML [17–21]. Unfortunately, Nguyen et al. [10] reported that immunotherapy did not successfully improve the OS of children with recurrent AML suggesting that immunotherapy might not be suitable for all children. To address this problem, we developed and verified an immune risk prognostic model according to seven immune genes to improve the precision treatment of AML-afflicted children. Additionally, this model could also predict the 5-year survival rate more accurately when compared to age, gender, and risk stratification.

In this study, the biological functions of DE immune genes were analyzed through bioinformatics. GO functional enrichment analysis showed that the DE genes were mainly enriched for receptor ligand activity, G protein−coupled receptor binding, leukocyte migration, external side of plasma membrane, regulation of hemopoiesis, cell chemotaxis, and leukocyte proliferation. These results support previous reports that abnormalities of these functions may lead to the progression of AML [22, 23]. The KEGG pathway was mainly enriched for cytokine-cytokine receptor related roles, JAK−STAT signaling pathway, MAPK signaling pathway, PI3K − Akt signaling pathway, and neuroactive ligand receptor related effects. Of note, continuous activation of the JAK−STAT signaling pathway and PI3K−Akt signaling pathway has been shown to contribute to the poor prognosis of AML [24–26]. Thus, the DE immune genes identified in this study may be responsible for the pathogenesis of AML.

On the basis of these DE immune genes, a prognostic risk model composed of seven immune genes, namely *GDF1*, *TPM2*, *IL1R1*, *PSMD4*, *IL5RA*, *DHCR24*, and *IL12RB2* was employed. The abnormal expression of *GDF1* is closely related to the poor prognosis of gastric cancer, liver cancer, and other tumors and *GDF1* is a member of the transforming growth factor beta superfamily (TGF-*β*) [27, 28]. A previous study showed that the expression of *GDF1* in poorly differentiated liver cancer was significantly increased and overexpression of *GDF1* inhibited cell proliferation but significantly increased tumor invasion and metastasis *in vivo* and *in vitro*. Meanwhile, overexpression of *GDF1* also enhanced the clinical therapeutic effects of cytotoxic T cell infiltration and immune checkpoint inhibitors in mouse models [28]. Taken together, this suggests that high expression of *GDF1* could enhance cancer patients' sensitivity to immunotherapy. Proteasome 26S subunit non-ATPase 4 (PSMD4) is an ubiquitin (UB) receptor involved in the degradation of ubiquitinated proteins and antigen processing. In addition, the expression of PSMD4 is abnormal in tumors such as colon cancer, liver cancer, breast cancer, and esophageal cancer [29–35]. Knocking out PSMD4 in MC38 colon cancer cells had no effect on cell proliferation but significantly weakened the immunotherapeutic effect of atractylenolide-I (ATT-I), which suggested that PSMD4 should play an important role in immunotherapy [36]. Deoxycholesterol reductase (DHCR24, also known as Seladin-1) is an enzyme in the final pathway of cholesterol synthesis and plays a crucial role in regulating cellular response to carcinogenesis and oxidative stress [37, 38]. DHCR24 is also overexpressed in bladder cancer, melanoma and endometrial cancer, prostate cancer, and breast cancer [39–42]. IL12RB2 is one of the subunits of the interleukin-12 (IL-12) receptor and its abnormal expression is closely related to the prognosis of laryngeal cancer [43, 44]. Airoldi et al. [45] identified lymph node plasmacytoma or lung cancer in IL12RB2 gene deficient mice, which indicated that targeted inactivation of *IL12RB2* gene, can induce tumorigenesis. IL12RB2 has also been shown to exert antitumor activity by regulating the function of IL-12 to affect the host immune system [46, 47]. Beta-tropomyosin (TPM2) is a member of the actin binding protein family and its cytoskeleton can be used as both a sensor and mediator of apoptosis [48, 49]. Some studies have shown that abnormal expression of TPM2 maybe closely associated with the development of colorectal cancer and breast cancer [50, 51]. Interleukin-1R1 which plays an important role in cancer-related inflammation through NF- *κ*B and MAP kinase pathways is the main receptor for interleukin-1*α* and interleukin-1*β* interacting with the agonist ligand IL-1 *α* and IL-1 *β* [52, 53]. Zhang et al. [53] reported that high expression of IL-1R1 in gastric cancer patients predicted a poor prognosis because of the over-activation of M2 macrophages and excessive infiltration of CD8 + T cells. At present, the role of IL5RA in tumorigeneisis is not known.

Although there are no previous reports on the association of the immune genes that make up our model, we have shown that these seven genes align well with the prognosis of AML patients within the entire cohort. Low expression of *IL5RA* and *GDF1* contributed to the poor prognosis of children with AML, while high expression of *TPM2*, *IL1R1*, *PSMD4*, *DHCR24*, and *IL12RB2* contributed to the poor prognosis of AML-afflicted children. The AUC of the prognostic model used to predict the 1-, 3- and 5-year survival rates was 0.701, 0.714 and 0.703, respectively, which suggested that this set of seven immune genes might provide higher specificity and sensitivity to predict the survival rates for AML patients. In addition, the nomogram constructed by the 7 immune genes in our model also proposed that the nomogram might have good predictive performance in terms of predicting the survival rate of the individual patient with AML, as confirmed by the calibration curve. Thus, our results support the proposal that those immune genes may reflect the progression of AML. Nonetheless, the specific mechanisms and pathways involving these gene products in the progression of AML still need to be unraveled through further studies.

To evaluate the accuracy of the prediction and clinical applicability of the model, several clinical variables and risk scores were determined in the training group, the test group, and the entire cohort. The analysis showed that age, risk stratification, CR2, and risk score were related to prognosis. Furthermore, risk stratification, CR2, and risk score were determined as independent prognostic variables. We also analyzed the relationship between immune genes and risk stratification in the model within the entire cohort and found that high expression of *IL5RA* and *GDF1* was associated with low-risk stratification, while high expression of *PSMD4*, *DHCR24*, and *IL12RB2* was associated with higher risk stratification. Our findings also concurred with previous reports, which demonstrated that age and risk stratification were closely related to the prognosis of AML patients [54–56]. Meanwhile, when analyzing the possible correlation between CR2 and the genes in the model, we found that expression levels of *IL5RA* and *GDF1* were lower in children with AML who did not go into remission even after the second course of treatment following recurrence. On the other hand, expression of PSMD4 was higher, suggesting that the model might be helpful to predict the OS of relapsed and refractory AML patients. In addition, this study further compared the predictive ability of several clinical variables with risk score. The results showed that the predictive value of our model in forecasting a 5-year OS was higher than the predictive value calculated when age, gender, risk stratification, and CR2 were used. Moreover, it is well known that some mutated genes such as *FIT3-ITD*, *WT1*, *CEBPA*, and *NPM1*, are closely related to prognosis. To further verify whether our model could estimate the prognosis of AML patients with those mutated genes, we divided the entire cohort into HR group and LR group based on the median value of risk score firstly, and then we analyzed their occurrence in each groups. It was observed that the probability of FIT3-ITD mutation and FIT3-ITD combined with WT1 mutation was remarkably higher in the HR group, whereas the probability of CEBPA and NPM1 mutation was markedly higher in the LR group (Supplementary Table 2). This indicated that our findings were agreement with the result of previous studies, which reported that if AML patients had FIT3-ITD or FIT3-ITD combined with a WT1 mutation, their prognosis was poor, while the prognosis was better when there was a CEBPA or NPM1 mutation [57]. Thus, our results show that the immune genes prognostic risk model we constructed has high predictive ability and is also reliable.

Previous evidence indicated that immune infiltration was a contributing factor in AML development and resistance to treatment [58, 59] and inhibition of CD8^+^ T cell function was conducive for tumor growth [60]. Impaired or inhibited CD8^+^ T cell function may cause AML cells to escape immune monitoring resulting in patient relapse [61, 62]. It has also been reported that when inhibited neutrophils take a longer time to recover in AML patients after chemotherapy, the possibility of AML recurring increases significantly [63]. It was previously reported that macrophage counts in AML patients were significantly higher than healthy individuals. Macrophages promote tumor cell proliferation, invasion, and metastasis while also inhibiting antitumor immune responses. Therefore, macrophages may play a positive role in the occurrence of AML [64, 65]. In this study, we showed that the risk score was negatively correlated with CD8^+^ T cell and neutrophil infiltration but positively correlated with macrophage infiltration, suggesting that the model might be able to reveal the immune microenvironment of AML patients. Collectively, we propose that our model is superior for predicting the prognosis of AML.

## 5. Conclusion

The prognostic risk model, which is also available to young AML, constructed in this study can predict the OS for children with AML more accurately and significantly reduces the need for whole genome sequencing of the patients. The nomogram constructed based on the developed model is expected to assist doctors in using a personalized medicine approach for the treatment of AML patients. However, our study is limited by the reliance on data within public databases and the prognostic model has not been verified using the additional external datasets as the GEO database related to childhood AML lacks survival data. In addition, this proposed model also has not been verified through clinical trials. In the future, we plan to conduct prospective trials in the clinic to verify the accuracy of the model and develop a kit for evaluating the prognosis of AML in the future to help doctors judge the prognosis and select the optimal treatment.

## Figures and Tables

**Table 1:** KEGG pathway enrichment analysis of DE genes in childhood AML patients.

Pathway ID	Description	*P* value	Gene count	Genes
hsa04060	Cytokine-cytokine receptor interaction	3.81E-54	109	*XCR1/OSM/CXCL12/BMP5/TNFRSF17/CXCR6/IL2RB/LEPR/GDF15/FASLG/PPBP/INHBE/INHBC/GHR/IL15/* *ACVR2A/CD70/PF4V1/IL18/GDF9/TNFRSF13B/IL2RG/GDF1/LIFR/GDF5/IL23A/IL10/IL1R2/IL11/IL1R1/* *TNFSF9/GDF11/CXCR3/IL18RAP/IL17B/IFNB1/CCR5/IL9R/IL17RE/EPOR/CX3CR1/TNFRSF8/IL1RL1/* *TNFRSF10D/IL5RA/TNFRSF6B/IL1RL2/ACVR1C/IL7R/CCL25/IL12A/CCR4/PF4/TNF/IL3RA/MSTN/CCR10/* *CCL1/IL33/CCR6/CCL28/GH1/BMP6/CTF1/EBI3/PRL/CSF3/IL13/INHBB/LTA/TSLP/BMP10/MPL/TNFRSF4/* *IL17D/CXCL8/TNFSF14/CCR1/IL2RA/IL24/IL17RB/GDF3/NODAL/IFNLR1/CXCL13/CXCL3/OSMR/CCL24/* *CCL27/NGFR/CXCL16/INHA/IL7/IL27/CRLF2/TNFRSF11A/IFNK/IL13RA2/BMPR1B/CXCL11/AMHR2/CSF2/* *ACKR4/IL12RB2/BMP2/CXCL5/IL12B/LEP/CXCL14*
hsa04630	JAK-STAT signaling pathway	5.75E-20	49	*OSM/PDGFRA/STAT5A/IL2RB/EGFR/LEPR/SOCS5/SOCS3/GHR/IL15/IL2RG/LIFR/IL23A/IL-10/HRAS/IL11/* *IFNB1/IL9R/EPOR/JAK3/IL5RA/IL7R/PDGFB/IL12A/IL22RA2/IL3RA/SOCS2/GH1/CTF1/PRL/CSF3/IL13/TSLP* */BCL2L1/MPL/IL17D/IL2RA/IL24/IFNLR1/OSMR/EGF/IL7/CRLF2/IFNK/IL13RA2/CSF2/IL12RB2/IL12B/LEP*
hsa04061	Viral protein interaction with cytokine and cytokine receptor	8.36E-17	35	*XCR1/CXCL12/IL2RB/PPBP/PF4V1/IL18/IL2RG/IL10/CXCR3/IL18RAP/CCR5/CX3CR1/* *TNFRSF10D/CCL25/CCR4/PF4/TNF/CCR10/CCL1/CCR6/CCL28/LTA/CXCL8/TNFSF14/CCR1/IL2RA/IL24/* *CXCL13/CXCL3/CCL24/CCL27/CXCL11/ACKR4/CXCL5/CXCL14*
hsa05133	Pertussis	2.78E-15	29	*NLRP3/RELA/TRAF6/MAPK3/CASP7/CD14/RHOA/NFKB1/GNAI2/NOD1/IL23A/IL10/IRF1/TLR4/C4B/C2/C1S/* *JUN/PYCARD/IL12A/TNF/TICAM1/C5/GNAI1/CXCL8/SERPING1/C4BPA/CXCL5/IL12B*
hsa04650	Natural killer cell mediated cytotoxicity	1.56E-13	36	*KLRD1/SH2D1B/GZMB/PRF1/FASLG/MAPK3/MICA/NCR1/MICB/HLA-A/KLRC1/FCGR3A/KLRK1/HRAS/* *HCST/FYN/KIR3DL1/IFNB1/HLA-C/RAET1E/TNF/KIR2DL3/RAC2/NCR2/RAC3/TYROBP/KIR2DL1/CD244/* *KIR3DL2/HLA-G/SHC4/SH2D1A/FCER1G/SHC3/ULBP1/CSF2*
hsa04612	Antigen processing and presentation	4.13E-13	27	*KLRD1/HSPA5/CALR/PDIA3/HSP90AB1/RFXANK/CTSS/CD8A/HLA-A/KLRC1/HSPA1B/TAPBP/KIR3DL1/PSME1/PSME2/HLA-C/HSP90AA1/HSPA1A/TNF/KIR2DL3/HSPA2/HSPA6/KIR2DL1/KIR3DL2/HLA-G/HLA-DMA/CD74*
hsa05417	Lipid and atherosclerosis	1.37E-12	46	*HSPA5/DDIT3/ATF4/NLRP3/HSP90AB1/RELA/TRAF6/XBP1/FASLG/MAPK3/CASP7/VCAM1/VLDLR/CD36/CD14/* *IL18/RHOA/NFKB1/APOA1/CAMK2A/HSPA1B/HRAS/TLR4/OLR1/IFNB1/CYBB/HSP90AA1/JUN/PYCARD/IL12A* */HSPA1A/RXRA/TNF/TICAM1/ABCA1/CASP6/HSPA2/HSPA6/BCL2L1/ABCG1/CXCL8/CXCL3/SELE/LBP/MMP9/IL12B*
hsa04151	PI3K-Akt signaling pathway	5.98E-11	59	*OSM/PDGFRA/PKN1/THBS1/KITLG/ATF4/HSP90AB1/RELA/IL2RB/EGFR/VEGFA/FGF17/FASLG/MAPK3/ATF2/GHR/FGFR4/FLT3/IL2RG/FGFR1/NFKB1/TNC/HRAS/TLR4/IFNB1/EIF4EBP1/EPOR/HSP90AA1/JAK3/VEGFC/IL7R/PDGFB/RXRA/FGF5/IL3RA/NR4A1/CDK6/GH1/ITGB3/* *PRL/CSF3/HGF/PDGFC/PGF/BCL2L1/FGF18/ANGPT1/FLT4/IGF1/IL2RA/OSMR/EGF/NGFR/IGF2/IL7/* *FGF10/VTN/ANGPT4/FGF2*
hsa04010	MAPK signaling pathway	9.65E-11	52	*XCR1/CXCL12/NFKBIB/RELA/CXCR6/CRKL/PPBP/MAPK3/PF4V1/RHOA/NFKB1/GNAI2/HRAS/GSK3A/CXCR3/* *CCR5/CX3CR1/JAK3/CCL25/CCR4/PF4/CCR10/CCL1/CCR6/CCL28/RAC2/RAC3/GNAI1/CXCL8/CCR1/SHC4/* *CXCL13/CXCL3/CCL24/CCL27/CXCL16/SHC3/CXCL11/CXCL5/CXCL14*
hsa04062	Chemokine signaling pathway	1.01E-10	40	*PKN1/NFKBIB/NLRP3/HSP90AB1/RELA/TRAF6/NLRP7/MAPK3/IL18/DEFA4/RHOA/NFKB1/NOD1/TLR4/NLRP6/* *DEFA3/NOD2/IFNB1/CYBB/DEFA1/MEFV/NAMPT/HSP90AA1/JUN/NLRP12/PYCARD/GABARAP/TNF/TICAM1/* *RIPK2/DEFA1B/CARD9/BCL2L1/CAMP/NLRC4/CXCL8/GBP7/CXCL3*

**Table 2:** A seven-gene signatures identified based on MCR.

Gene ID	Coef	Exp (coef)	Se (coef)	Z	Pr(>|z|)
*GDF1*	-0.07435	0.928345	0.02859	-2.60065	0.009305
*TPM2*	0.279473	1.322432	0.052832	5.289841	1.22E-07
*IL1R1*	-0.10441	1.110055	0.034104	3.061558	0.002202
*PSMD4*	0.250769	1.285013	0.080676	3.108343	0.001881
*IL5RA*	-0.06511	0.936968	0.027078	-2.40437	0.0162
*DHCR24*	0.129822	1.138626	0.034274	3.78782	0.000152
*IL12RB2*	0.068248	1.070631	0.021656	3.151412	0.001625

## Data Availability

The transcriptome data sets in my article were downloaded from the Target database (https://ocg.cancer.gov/programs/target) which is a public database.

## Supplementary Materials

Supplementary Materials Supplementary Table 1: the 114 DE immune genes screened with univariate Cox regression and Kaplan-Meier analyses. Supplementary Table 2: evaluate the prognostic model by mutation genes of FLT3-ITD, WT1, CEBPA, and NPM1.
