# Supplementary material for: A Prognostic Model of Seven Immune Genes to Predict Overall Survival in Childhood Acute Myeloid Leukemia
Source: Biomed Res Int. 2022 Dec 5;2022:7724220. doi: 10.1155/2022/7724220 (PMC9744619; doi:10.1155/2022/7724220)
Supplement: Supplementary Materials — Supplementary Table 1: the 114 DE immune genes screened with univariate Cox regression and Kaplan-Meier analyses. Supplementary Table 2: evaluate the prognostic model by mutation genes of FLT3-ITD, WT1, CEBPA, and NPM1. [file 7724220.f1.docx]

Supplementary Materials for

A prognostic model of seven immune genes to predict overall survival in childhood Acute Myeloid Leukemia

Yan Luo ^1,2,3,^ Yanpeng Xu ^2,3^, Xue Li ^2^, Xiaoqi Shi ^2^, Pei Huang ^2,3^, Yan Chen ^2^*, Zhixu He ^2,3,4^*,

1 Suzhou Medical College of Soochow University, Suzhou, 215325, China

2 Department of Pediatrics, Affiliated Hospital of Zunyi Medical University, Zunyi 563000, China

3 Department of Pediatrics, Guizhou Children's Hospital, Zunyi 563000, China

4 Collaborative Innovation Center of Tissue Damage Repair and Regeneration Medicine of Zunyi Medical University, Zunyi 563003, Guizhou, China

* Corresponding author: Zhixu He, Professor, Chief physician; E-mail: [hzx@gmc.edu.cn](mailto:hzx@gmc.edu.cn). Yan Chen, Professor, Chief physician; E-mail: [cyz600@163.com](mailto:cyz600@163.com).

Supplementary Table 1: The 114 DE immune genes screened with univariate Cox regression and Kaplan-Meier analyses

| Gene | HR | z | pvalue | K-M Pvalue |
| --- | --- | --- | --- | --- |
| *DAGLB* | 0.64862071 | -5.711117464 | 1.12E-08 | 6.11E-05 |
| *NXN* | 1.148056635 | 5.289923756 | 1.22E-07 | 1.65E-04 |
| *MPO* | 0.914508253 | -5.048198867 | 4.46E-07 | 1.22E-06 |
| *IL12RB2* | 1.109633051 | 4.967254987 | 6.79E-07 | 1.23E-04 |
| *PDK1* | 0.723621127 | -4.592483057 | 4.38E-06 | 1.69E-05 |
| *HDAC11* | 1.401203521 | 4.561927287 | 5.07E-06 | 1.55E-03 |
| *NEO1* | 1.103085042 | 4.412980618 | 1.02E-05 | 9.68E-04 |
| *TPM2* | 1.282576831 | 4.391671304 | 1.12E-05 | 4.91E-03 |
| *BMP2* | 1.106451638 | 4.387339829 | 1.15E-05 | 3.29E-03 |
| *RORB* | 1.128601398 | 4.30686689 | 1.66E-05 | 4.19E-02 |
| *FGFR1* | 0.862838334 | -4.194006556 | 2.74E-05 | 5.81E-04 |
| *IL5RA* | 0.902917209 | -4.174766743 | 2.98E-05 | 1.51E-06 |
| *CALR* | 0.730453392 | -4.151685084 | 3.30E-05 | 9.84E-04 |
| *TMEM173* | 1.315490616 | 4.116485197 | 3.85E-05 | 5.16E-04 |
| *DHCR24* | 1.148055356 | 4.106950157 | 4.01E-05 | 6.57E-04 |
| *SEMA4G* | 1.144131543 | 4.087791634 | 4.35E-05 | 3.79E-04 |
| *TPSB2* | 0.931345781 | -4.085466534 | 4.40E-05 | 2.08E-05 |
| *RARB* | 1.108469444 | 4.044913052 | 5.23E-05 | 1.02E-04 |
| *TRH* | 0.941944372 | -3.999977402 | 6.33E-05 | 3.06E-03 |
| *BST2* | 1.189014996 | 3.94464751 | 7.99E-05 | 9.01E-04 |
| *HGF* | 0.918644158 | -3.902274733 | 9.53E-05 | 1.27E-04 |
| *SERPING1* | 1.117332376 | 3.879251541 | 0.000104778 | 2.78E-05 |
| *SYP* | 1.193567816 | 3.866870266 | 0.000110241 | 4.17E-04 |
| *CETP* | 0.898951506 | -3.841908423 | 0.000122081 | 2.49E-04 |
| *SOCS2* | 1.100777358 | 3.841650493 | 0.00012221 | 1.04E-03 |
| *FLT4* | 0.918693627 | -3.835113872 | 0.000125506 | 1.48E-02 |
| *SEMA5B* | 1.126657975 | 3.700963704 | 0.000214782 | 8.48E-03 |
| *PDGFC* | 0.917632524 | -3.682098843 | 0.000231322 | 3.69E-07 |
| *SEMA3A* | 1.093109418 | 3.677063139 | 0.000235935 | 2.55E-03 |
| *UMODL1* | 0.916552514 | -3.665842249 | 0.000246526 | 9.93E-05 |
| *ITGB3* | 1.0980397 | 3.636561121 | 0.000276302 | 1.49E-03 |
| *IL1RL1* | 0.927365518 | -3.590684266 | 0.000329811 | 6.03E-03 |
| *GPI* | 0.77187044 | -3.55514888 | 0.000377765 | 1.33E-03 |
| *STAT5A* | 0.732644928 | -3.521303292 | 0.000429431 | 2.46E-02 |
| *NPR3* | 0.931494263 | -3.50975586 | 0.000448518 | 1.19E-03 |
| *KLK1* | 1.106826236 | 3.479288199 | 0.000502748 | 5.63E-03 |
| *TRIM47* | 0.893957545 | -3.469265791 | 0.000521883 | 1.28E-02 |
| *PRDX1* | 1.180464704 | 3.429435199 | 0.000604839 | 1.91E-03 |
| *TMSB15B* | 1.137091658 | 3.35124153 | 0.000804501 | 7.63E-03 |
| *TLR9* | 1.195436026 | 3.291377338 | 0.000996981 | 1.27E-02 |
| *RANBP9* | 0.721772337 | -3.280495177 | 0.00103625 | 6.60E-03 |
| *PTH2R* | 0.930259539 | -3.247624756 | 0.001163726 | 4.72E-03 |
| *RBP5* | 1.096620335 | 3.213630164 | 0.001310684 | 4.49E-03 |
| *DUOX1* | 1.077978008 | 3.203289054 | 0.001358675 | 9.17E-03 |
| *FSTL1* | 1.088975721 | 3.172390246 | 0.001511897 | 6.82E-04 |
| *ROBO3* | 1.125286426 | 3.152375887 | 0.001619477 | 9.55E-03 |
| *IL7* | 1.08779667 | 3.147042923 | 0.001649307 | 2.01E-03 |
| *PDIA3* | 0.762077773 | -3.127250409 | 0.001764496 | 3.44E-02 |
| *ELF1* | 0.767650002 | -3.081361251 | 0.002060565 | 2.82E-02 |
| *CNOT8* | 0.73982111 | -3.047535955 | 0.002307259 | 4.13E-02 |
| *RIPK2* | 0.848342234 | -3.044617372 | 0.002329765 | 7.71E-04 |
| *SIAH1* | 0.811435616 | -3.039372892 | 0.002370712 | 7.42E-03 |
| *FGF2* | 1.076979378 | 3.038707484 | 0.002375954 | 8.14E-03 |
| *NLRP7* | 1.117871113 | 3.02183458 | 0.002512478 | 4.49E-03 |
| *BMPR1B* | 1.06724692 | 2.999852417 | 0.002701104 | 4.43E-05 |
| *GDF1* | 0.91927329 | -2.983312202 | 0.00285147 | 1.67E-03 |
| *MMP7* | 1.082364227 | 2.973781467 | 0.002941545 | 7.64E-04 |
| *NFAT5* | 0.831856089 | -2.942847347 | 0.003252087 | 4.71E-02 |
| *PGLYRP2* | 1.104014076 | 2.933635768 | 0.003350171 | 3.92E-04 |
| *VEGFC* | 1.082861165 | 2.908612792 | 0.003630362 | 9.33E-04 |
| *NTN1* | 0.927723864 | -2.904892031 | 0.003673798 | 4.34E-03 |
| *ILF3* | 0.746692104 | -2.849642865 | 0.004376834 | 1.31E-03 |
| *NR5A2* | 0.922731727 | -2.847522246 | 0.004406101 | 2.63E-03 |
| *PF4* | 1.059344375 | 2.844635817 | 0.004446223 | 2.20E-03 |
| *PSMD4* | 1.242605693 | 2.842685511 | 0.004473519 | 4.41E-03 |
| *CXCL3* | 1.06764912 | 2.840036738 | 0.004510834 | 1.07E-02 |
| *MICB* | 0.772159956 | -2.827685651 | 0.004688582 | 4.25E-02 |
| *S100P* | 0.929365256 | -2.823090791 | 0.00475631 | 1.73E-02 |
| *IRF1* | 0.845461087 | -2.821994571 | 0.004772599 | 2.30E-02 |
| *VEGFA* | 0.890554616 | -2.817432974 | 0.004840922 | 5.20E-03 |
| *SMAD6* | 1.106920304 | 2.769627066 | 0.005612051 | 4.31E-02 |
| *CXCL5* | 1.069897718 | 2.761254418 | 0.00575798 | 1.35E-03 |
| *NUMBL* | 1.189808133 | 2.7408428 | 0.006128182 | 3.31E-02 |
| *CTSS* | 0.856710285 | -2.721952066 | 0.006489755 | 2.76E-03 |
| *MAPKAPK2* | 0.815893004 | -2.711198054 | 0.006704057 | 9.34E-03 |
| *TSLP* | 1.061505934 | 2.707296392 | 0.006783367 | 6.79E-03 |
| *ELANE* | 0.948715266 | -2.697335 | 0.006989691 | 4.41E-02 |
| *TPP2* | 0.812031933 | -2.686130719 | 0.00722848 | 8.99E-03 |
| *CD200* | 0.926945261 | -2.662437251 | 0.007757703 | 4.71E-03 |
| *KLRK1* | 0.890830189 | -2.649491078 | 0.00806131 | 1.18E-02 |
| *PSEN2* | 1.127703686 | 2.645994663 | 0.00814511 | 3.59E-02 |
| *S100Z* | 0.942242707 | -2.624838533 | 0.008669007 | 2.28E-02 |
| *NR2C1* | 0.785551512 | -2.621957859 | 0.008742624 | 2.32E-02 |
| *IFI6* | 1.086688205 | 2.60616193 | 0.009156316 | 3.60E-03 |
| *NCR2* | 1.056052544 | 2.573400277 | 0.010070466 | 1.17E-02 |
| *HSPA14* | 0.755160505 | -2.548137851 | 0.010829967 | 9.86E-03 |
| *UCN* | 1.160451126 | 2.516514573 | 0.0118522 | 2.60E-02 |
| *SNCA* | 1.081601749 | 2.513571461 | 0.011951556 | 6.71E-03 |
| *DUSP10* | 0.8663869 | -2.497329711 | 0.012513255 | 4.55E-02 |
| *TRIM13* | 0.808663789 | -2.478054415 | 0.0132101 | 3.87E-02 |
| *NRTN* | 1.089140887 | 2.459336639 | 0.013919403 | 3.04E-02 |
| *BAIAP2L1* | 1.068805765 | 2.448498597 | 0.014345299 | 5.93E-03 |
| *ELN* | 1.057403591 | 2.427086506 | 0.015220626 | 9.82E-03 |
| *RELB* | 0.88353442 | -2.425474311 | 0.015288398 | 9.82E-03 |
| *NGFR* | 1.079226543 | 2.39811328 | 0.016479768 | 1.19E-02 |
| *APOBEC3H* | 1.10737076 | 2.362502453 | 0.018152021 | 1.73E-02 |
| *LST1* | 1.079673992 | 2.328226455 | 0.019900082 | 6.21E-03 |
| *MSTN* | 1.089850797 | 2.323154099 | 0.020170876 | 3.10E-02 |
| *MIA* | 1.095438861 | 2.288051917 | 0.022134498 | 1.64E-02 |
| *PKLR* | 0.945649831 | -2.287144363 | 0.022187398 | 1.42E-03 |
| *CALCOCO2* | 0.824090325 | -2.234852115 | 0.025427059 | 7.96E-03 |
| *NOD1* | 0.850505972 | -2.189901286 | 0.028531397 | 2.51E-02 |
| *PCBP2* | 0.793439403 | -2.183252102 | 0.02901725 | 1.43E-02 |
| *APOA1* | 1.067855241 | 2.169574968 | 0.030039059 | 3.63E-03 |
| *TRPC4AP* | 0.781911966 | -2.167591767 | 0.030189761 | 1.00E-02 |
| *IL1R1* | 1.069664421 | 2.1028034 | 0.035482959 | 4.89E-02 |
| *ECSIT* | 1.19210852 | 2.065308837 | 0.038893788 | 2.46E-02 |
| *GLI1* | 1.079360591 | 2.023530611 | 0.043018471 | 4.99E-02 |
| *SOCS5* | 0.840171784 | -1.999713891 | 0.045531167 | 3.29E-02 |
| *FYN* | 1.132776573 | 1.995748615 | 0.045961293 | 1.24E-02 |
| *PSME2* | 1.148364574 | 1.989191759 | 0.046680041 | 3.22E-02 |
| *VLDLR* | 0.959560362 | -1.98182371 | 0.047498975 | 4.72E-02 |
| *TLR5* | 1.057682049 | 1.978292226 | 0.04789575 | 2.05E-02 |
| *GDF11* | 1.086735886 | 1.974470275 | 0.048328294 | 1.94E-02 |

Supplementary Table 2: Evaluate the prognostic model through mutation genes of FLT3-ITD, WT1, CEBPA and NPM1

|  | High risk (n=558) | Low risk (n=559) | *P*-value |
| --- | --- | --- | --- |
| FLI3-ITD mutation | 114 (20.43%) | 78 (13.95%) | 0.004 |
| FLI3-ITD combined with WT1 mutation | 12 (2.15%) | 1 (0.18%) | 0.02 |
| CEBPA mutation | 15 (2.69%) | 56 (10.02%) | <0.0001 |
| NPM mutation | 41 (7.35%) | 61 (10.91%) | 0.038 |
